# Neutralizing activities of caprine antibodies towards conserved regions of the HCV envelope glycoprotein E2

**DOI:** 10.1186/1743-422X-8-391

**Published:** 2011-08-05

**Authors:** Yasmine S El Abd, Ashraf A Tabll, Noha G Bader El Din, Alaa El-Dien S Hosny, Rehab I Moustafa, Reem El-Shenawy, Khaled Atef, Mostafa K El-Awady

**Affiliations:** 1 Department of Microbial Biotechnology, National Research Center, Giza, Egypt; 2 Microbiology and Immunology Department, Faculty of Pharmacy, Cairo University, Egypt

**Keywords:** Hepatitis C virus (HCV), anti E2 antibodies, neutralizing antibodies, *In vitro *culture model for HCV, candidate peptide vaccine for HCV

## Abstract

Anti HCV vaccine is not currently available and the present antiviral therapies fail to cure approximately half of the treated HCV patients. This study was designed to assess the immunogenic properties of genetically conserved peptides derived from the C-terminal region of HVR-1 and test their neutralizing activities in a step towards developing therapeutic and/or prophylactic immunogens against HCV infection. Antibodies were generated by vaccination of goats with synthetic peptides derived from HCV E2. Viral neutralizing capacity of the generated anti E2 antibodies was tested using in vitro assays. Goats immunized with E2 synthetic peptides termed p412 [a.a 412-419], p430 [a.a 430-447] and p517 [a.a 517-531] generated high titers of antibody responses 2 to 4.5 fold higher than comparable titers of antibodies to the same epitopes in chronic HCV patients. In post infection experiments of native HCV into cultured Huh7.5 cells anti p412 and anti p 517 were proven to be neutralizing to HCV genotype 4a from patients' sera (87.5% and 75% respectively). On the contrary anti p430 exhibited weak viral neutralization capacity on the same samples (31.25%). Furthermore Ab mixes containing anti p430 exhibited reduced viral neutralization properties. From these experiments one could predict that neutralization by Abs towards different E2-epitopes varies considerably and success in the enrichment of neutralization epitope-specific antibodies may be accompanied by favorable results in combating HCV infection. Also, E2 conserved peptides p517 and p412 represent potential components of a candidate peptide vaccine against HCV infection.

## Introduction

Hepatitis C Virus (HCV) is a global health problem that affects almost 3% of the world's population [1] and not less than 15% of the Egyptian population [2]. Individuals with chronic HCV infection usually remain asymptomatic and undiagnosed for decades before chronic hepatitis leads to severe fibrosis and cirrhosis, hepatic failure, or hepatocellular carcinoma [3-7]. These long-term complications made HCV one of the leading emerging infectious diseases worldwide. The current antiviral regimen, a combination of pegylated interferon α and ribavirin, is curative in about half of treated patients depending on the viral and/or host factors. Additionally, this regimen is expensive, requires prolonged therapy, sometimes with serious side effects and only a fraction of those with chronic HCV infections meet the criteria for treatment [8].

Viral proteins are recognized as non-self by the host's immune system and induce the production of antibodies. During the natural course of infection, a large number of antibodies are produced. The vast majority of antibodies induced have no antiviral activity, either because they are elicited by degraded or incompletely processed proteins released from dying cells or because they are directed against epitopes that do not play any role in the virus entry process "non-neutralizing antibodies". A small proportion of antibodies termed "neutralizing antibodies" are able to target exposed epitopes of the viral structural proteins and neutralize the infectious virus by preventing or controlling viral infection [9,10]. During the chronic phase of HCV infection, most HCV-infected patients develop high-titer of antibodies. Paradoxically, these antibodies were not able to control HCV infection which may be attributed to the generation of non-neutralizing HCV-specific antibodies that compete with neutralizing Abs and reduce their effectiveness. Such antibodies have been reported in other viral infections in which highly immunogenic non-neutralizing epitopes mislead the humoral immune response contributing to viral escape from neutralization [11]. Several observations support the hypothesis that neutralizing antibodies may help control HCV replication [12,13].

Synthetic peptide based vaccines were shown to generate specific Abs capable of neutralizing HCV infections [14,15]. In the present study, we utilized large scale multiple sequence alignment of E2 to design genetically conserved peptides from viral envelop proteins (particularly among type 4 isolates predominant in Egypt). The aim of this work is to develop monospecific polyclonal Abs in caprines against the 3 chosen conserved peptides derived from E2 glycoprotein and to test the immunogenic and viral neutralizing properties of each Ab using assays depending on blocking of viral infectivity to hepatoma cell line. Based on the obtained results, p412 and p517 represent candidate peptides for further assessment as potential therapeutic/prophylactic immunogens.

## Materials and methods

### Approval ethics

This research was approved by the Review Board of National Research Center, Egypt

### Design and synthesis of HCV E2 conserved peptides

Three peptides were designed and synthesized as previously described [16]. Peptides were all derived from the C-terminal region of HVR-1 and designated p412 [a.a 412-419], p430 [a.a 430-547] and p517 [a.a 517-531].

Immunization of caprines, production and purification of polyclonal antibodies

Six goats were immunized with either of the synthetic peptides p412, p 430 or p517 (2 animals for each peptide). Two goats were injected with 2 ml saline solution at the same time intervals of immunization protocol to serve as controls. The immunizing doses/goat were 1.5 mg of the peptide. Each linear peptide was emulsified with equal volume of Freund's complete adjuvant and was injected subcutaneously in three different sites within the same animal. On days 15 and 28, blood samples were collected and then each goat was reimmunized with a second booster dose of the same peptide emulsified with equal volume of Freund's incomplete adjuvant. The goats were sacrificed after 96 days from the last injection and the blood was collected to quantify the titer of relevant immunoglobulins. Caprine IgG was purified out following a procedure described by Mckinney and Parkinson, [17]. Briefly, IgG purification was carried out in two steps, step 1 involves elimination of albumin and other non IgG proteins via precipitation with Caprilyic acid (Octanoic acid; Riedel-DE HAEN AG, Seelze-Hannover, Germany). While step 2 includes further purification of IgG fractions using ammonium sulphate cut as reported by Mohanty and Elazhary [18].

### Efficacy and specificity of purified caprine IgGs towards the relevant E2 antigens in HCV infected human sera

Western blot analysis was used to detect circulating E2 proteins and/or relevant epitopes on HCV virions using the purified caprine antibodies as previously described [16,19,20]. In brief, strips with HCV serum proteins were washed 3 times and incubated with diluted first anti p412 Ab (1:100 in PBS-0.3% T20) at room temperature for 2 h. After 3 washes, strips were incubated with anti-caprine IgG peroxidase labeled second Ab (KPL, Gaithersburg, USA) diluted in 1: 5000 PBS-0.3% T for 2 h at room temperature. Visualization of bands on the nitrocellulose membrane was done by incubating the strips with 0.01 M PBS, pH 7.4 containing 50 mg, peroxidase specific substrate, diaminobenzedine DAB (Sigma; Deisenhofen, Germany) and 100 μl of 3% hydrogen peroxide.

### Establishment of cell culture system infected with HCV

#### Cell line

Human well differentiated hepatocellular carcinoma cell line Huh7 was modified to produce a more efficient cell line Huh7.5 a generous gift from Charles Rice (The Rockefeller University, USA). The cells were obtained under Material Transfer Agreement (MTA# 642 to Dr. Mostafa K. El Awady 2007). Huh7.5 cells were used for HCV infection experiments and assessment of viral replication. Huh7.50 cells were cultured and infected with HCV particles according to protocols described by Seipp et al., [21] and Song et al.,[22]. Assessment of the successful viral infection in Huh7.5 cells throughout the culture duration was confirmed at the transcriptional level by Reverse Transcription Polymerase Chain Reaction (RT-PCR) amplification of HCV RNA and at the translational level by SDS-PAGE of cell lysates followed by Western blotting to determine the de novo synthesis of non structural proteins e.g NS5A. Western blot was done as described before except that the membrane was incubated with diluted mouse monoclonal anti-NS5A (Ab, Abcam; Cambridge, UK) (1: 200 in PBS-0.3% T) at room temperature for 2 h and after the 3 washes, the membrane was incubated for 2 h at room temperature with diluted peroxidase labeled anti-mouse IgG Ab (1: 1000 in PBS-0.3% T). For Reverse transcription -PCR (RT-PCR), retrotranscription was performed in 25 μl reaction mixture containing 20 units of AMV reverse transcriptase (Clonetech, USA) with 400 ng of total cellular RNA as template, 40 units of RNAsin Clontech, USA), a final concentration of 0.2 mmol/l from each dNTP (Promega, Madison, WI, USA) and 50 pmol of the reverse primer P1. The reaction was incubated at 42°C for 60 min. and denatured at 98°C for 10 min. Amplification of the highly conserved 5'-UTR sequences was done using two rounds of PCR with 2 pairs of nested primers. The first round amplification was done in 50 μl reaction containing 50 pmol from each of P2 forward primers and P3 reverse primers, 0.2 mmol/l from each dNTP, 10 μl from RT reaction mixture as template and 2 units of Taq DNA polymerase (Promega, USA) in 1 × buffer supplied with the enzyme. The thermal cycling protocol was as follows: 1 min at 94°C, 1 min at 55°C and 1 min at 72°C for 30 cycles. The second round amplification was done as the first round, except for use of the nested reverse primer P4 and forward primer P5 at 50 pmol each. A fragment of 172 bp was identified in positive samples. Primer sequences are as follows: P1: 5'ggtgcacggtctacgagacctc 3'; P2: 5' aactactgtcttcacgcagaa 3'; P3: 5' tgctcatggtgcacggtcta 3'; P4: 5' actcggctagcagtctcgcg 3'; P5: 5' gtgcagcctccaggaccc 3'

### Testing the immunoreactivity of caprine anti p412 antibody with intracellular E2 protein

Intracellular expression of E2 antigen in infected Huh7.5 cells was confirmed via 3 independent immuno assays; namely western blotting, intracellular immunosaining and FACS analysis. Western blotting was performed as previously mentioned. Intracellular HCV immunostaining assay was achieved as follows: Cells (either infected or non infected) were grown in 6 well cell culture plates (Greiner Bio-one, Germany) for 48 h to semi-confluency. Prior to staining, cells were washed with PBS and immunostained according to a procedure described by Harlow and Lane [23]. Briefly, cells were fixed with 4% paraformaldehyde solution in PBS for 10 min at room temperature and then washed twice with PBS. Fixed cells were permeabilized by incubation in 0.2% TritonX-100 in PBS for 15 min, followed by washing 3 times with PBS. Blocking the non-specific antibody binding sites was done by incubating cells for 10 min in blocking buffer (PBS-1% BSA). Cells were incubated with anti p412 Ab at dilution 1/200 in PBS -1% BSA for 1 h at room temperature. Cells were then washed 3 X with PBS, 10 min each wash. Horseradish peroxidase conjugated anti-caprine IgG (KPL, Gaithersburg, USA) at dilution 1/2000 in PBS-1% BSA was added to the monolayer cells and incubated for 1 h at room temperature. Colored foci were revealed by incubating the cells with 0.01 M PBS, pH 7.4 containing 50 mg, peroxidase specific substrate, diaminobenzedine (Sigma; Deisenhofen, Germany) and 100 μl of 3% hydrogen peroxide and stopped after 10 min of incubation with distilled water. The distinct colored foci were examined and some positive and negative fields were photographed on phase contrast microscope (Olympus IX70, USA). To further analyze the immunoreactive properties of caprine anti p412 Ab antibody with the E2 antigen localized on the surface of Huh7.5 cells, FACS analysis of HCV E2 antigen was performed on surface of infected Huh7.5 cells as follows: cells were infected with a pool of high HCV viral loads. The plates were incubated at 37°C with media change every two days. After one week incubation, media were aspirated and cells were washed three times with PBS, collected then centrifuged and supernatants were removed. Cell pellets were washed 4 times with PBS. Surface labeling was performed by indirect immuno-fluorescence, where cells were incubated with anti p412 Ab (at dilution 1/200 in PBS) for 1 h at 4°C, and washed 4 times with PBS. Fluorescein isothiocyanate (FITC) labeled anti Caprine IgG (KPL, Gaithersburg, USA) at dilution 1: 2000 in PBS was added to the cells for 30 min at 4°C. Cells were washed 3 times with PBS containing 1% normal caprine serum to decrease nonspecific fluorescence, then cells were suspended in 500 μl PBS and analyzed with BD FACS Calibur™ Flow Cytometer (Becton Dickinson, USA). Mean fluorescence intensities were determined using cell Quest software (Becton Dickinson, USA).

### Testing the viral neutralizing capacity of caprine anti E2 antibodies

Viral neutralizing activity of caprine anti E2 antibodies was studied using HCV infected Huh7.5 and pursuing the viral replication via RT-PCR amplification. In order to study the antibody mediated neutralization, different concentrations of the generated E2 antibodies were pre-incubated with a pool of 10 HCV genotype 4a infected sera with viral loads ranging from 9 × 10^5^-1.5 × 10^6 ^copies/ml prior to incubation with Huh7.5 cells. Viral binding/entry to cells was detected by nested RT-PCR. In a 1.5 ml tube, HCV positive sera that were pre-diluted with FCS free media (1: 50) was incubated with serial dilutions of caprine anti E2 antibodies from 150 μg/ml to 2.5 μg/ml in a final volume of 1 ml FCS-free DMEM. Pooled HCV positive sera as well as HCV negative (negative HCV RNA and IgG Abs) were diluted 1:50 in 1 ml FCS-free DMEM and used for infection assays as positive and negative controls respectively. All tubes were then incubated overnight at 4°C before supplementation to cells. Huh7.5 cells (200,000 cells/well) were grown to semi-confluence in DMEM supplied with 10% FCS, 1% Penicillin-Streptomycin and 0.1% fungizone in 6 well plates for 48 h. The media were aspirated, then cells were washed twice with FCS-free medium and inoculated with 1 ml of the previously incubated sera-antibody mix. For each assay, two wells were left without serum inoculation (supplied only with DMEM) to serve as reagent negative controls. After 90 minutes at 37°C in 5% CO_2_, 1 ml DMEM containing 10% FCS was added to each well. On the next day media were aspirated and cells were washed three times with PBS to get rid of the remaining sera-antibody mix, replaced with fresh medium (DMEM-10% FCS) and cells were further incubated for 24 h at 37°C. On the third day, media were aspirated and 500 μl of (4 M guanidinium thiocyanate/0.5% sarcosyl) was added to each well. Extraction of total cellular RNA was done as described by El Awady et al [19]. Amplification of of HCV RNA was performed using 5'UTR specific nested primers as stated above.

### Effect of HCV viral load on infectivity of Huh7.5 cultured cells and on the blocking activity of the generated caprine antibodies

To test whether the viral load in HCV positive samples affects viral entry into Huh7.5 cells or the blocking activity of the generated caprine antibodies, viral loads were quantified in 20 samples prior to application in the infection experiments. A statistical correlation was made between viral loads in each sample and the blocking activity of the generated caprine antibodies. T- Test was conducted to correlate both parameters. Testing the blocking of viral entry into hepatoma cells by different combinations of caprine anti E2 antibodies

To test the possible molecular and/or steric interference of a combination of two caprine anti E2 antibodies on blocking of viral entry into cultured Huh7.5 cells, a pool of 10 HCV 4a positive sera were pre-incubated with the following 3 Ab combinations (anti p412 + anti p430), (anti p412 + anti p517) and (anti p430+ anti p517) prior to infection of cells. Viral RNA was extracted and subjected to intracellular viral evaluation using both nested RT-PCR as previously described and quantitatively by Real Time PCR (Qiagen, CA USA), where a single-tube real time RT-PCR assay was performed for the quantification of the 5' NCR of HCV by using Taqman technology [Roche Molecular Diagnostics Systems].

### Statistical analyses

All statistical analyses were performed using the SPSS 9.0 statistical software program. The difference is considered statistically significant when p ≤ 0.05.

## Results

### Specificity of purified caprine IgG in detection of circulating E2 antigen in HCV positive sera

**Figure 1:**
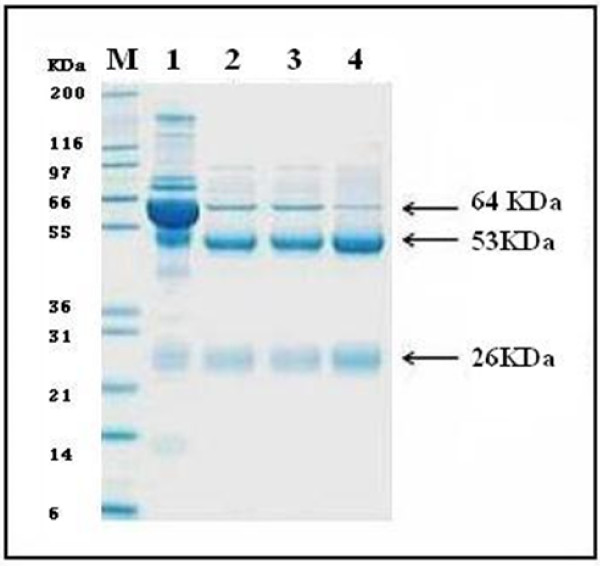
**Two step partial purification of caprine IgG's from E2 synthetic peptide immunized caprines**. Caprine sera before (lane 1) and after purification of p412, p430 and p517 (lanes 1, 2 and 3 respectively) immunized caprine sera were resolved on SDS PAGE followed by Coomassie blue staining. The caprine albumin appears as a dense band at ~ 64 kDa in unpurified serum and much less dense in purified sera. Lanes 1, 2 and 3 show distinct bands of heavy and light chain IgG at 53 and 26 kDa after purification. Lane M represents the Broad Range Protein Molecular Weight Marker (Promega; Madison, USA).

**Figure 2:**
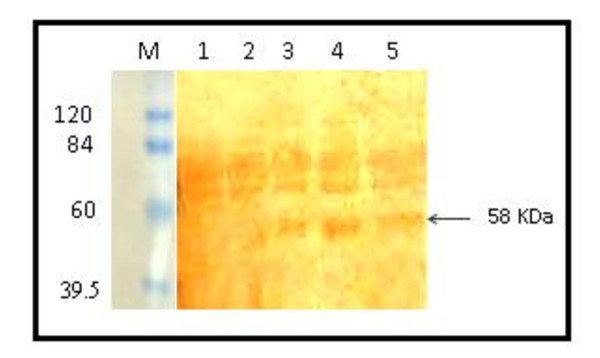
**Western blott analysis showing the ability of the generated caprine antibodies to recognize the E2 protein in HCV infected sera**. After SDS-PAGE on 12% gels under non reducing conditions, resolved proteins were electrically transferred into nitrocellulose membranes and enzyme immune reaction was developed. Lanes 1 and 2 represent sera of healthy individuals as negative controls. Lanes3, 4 and 5 represent sera from three HCV infected patients. A band of ~ 58 kDa was detected only in HCV positive but not in healthy sera. M lane represents the molecular weight protein marker.

Figure 1 shows the purified IgG that was subsequently used in western blot analysis. The resolved HCV proteins from infected patients were electrotransferred onto nitrocellulose membrane and allowed to react with purified IgG from p412 immunized goats. A discrete band at ~ 58 kDa appeared only in HCV positive sera but could not be detected in healthy sera (Figure 2) indicating the presence of a circulating HCV specific antigen possibly E2 in infected serum samples. ELISA and Dot EIA assays demonstrated a high binding affinity of the generated IgG's towards the corresponding synthetic peptides in a peptide specific fashion as previously shown in El Awady *et al*., [16] indicating the absolute specificity of IgG epitope interaction.

### Evaluation of Huh7.5 hepatoma cells' support of HCV replication *in vitro*

**Figure 3:**
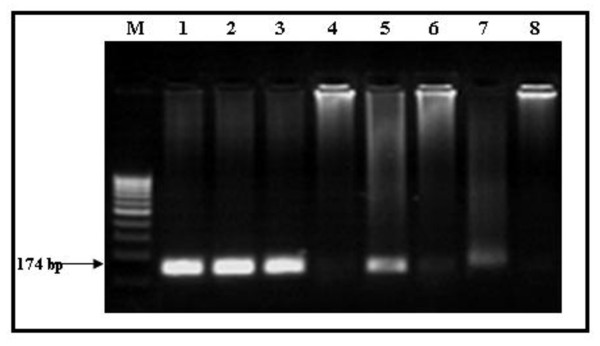
**Agarose gel electrophoresis of nested RT-PCR products to evaluate HCV infection into Huh7.5 cells from HCV positive sera**. The HCV nested RT-PCR products at 174 bp band were visualized on 2% agarose gels stained with ethidium bromide. Lane 1: viral positive strand in the HCV genotype 4 patients' sera. Lane 2: viral positive strand in the patients' PBMCS. Lane 3: negative strand in the patients' PBMCS. Lane 4: nested RT-PCR product of the last wash of Huh7.5 cells to make sure there isn't ny residual virus from the infectious serum. Lane 5: viral positive strand in the cells 3 days post infection. Lane 6: viral negative strand in the cells 3 days post infection. Lane 7: viral positive strand in the supernatant 3 days post infection. Lane 8: viral negative strand in the cells' supernatant. M: molecular weight standard DNA marker (100 base pair; Jena Bioscience, Germany).

Direct infection of Huh7.5 cells from positive HCV sera resulted in a detectable intracellular levels of both genomic (Figures 3 and 10b) and antigenomic (Figure 3) strands thus suggesting intracellular genomic replication of HCV. Western blot analysis to test the expression of viral NS5A (non structural) protein in lysates of infected Huh7.5 cells

**Figure 4:**
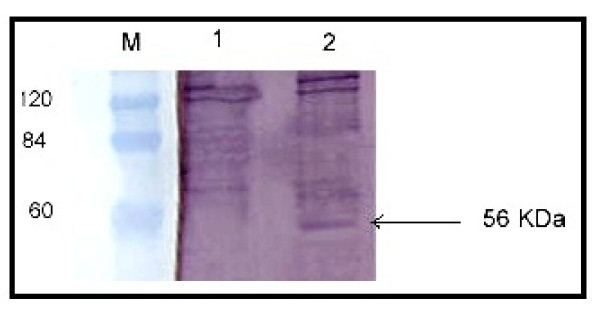
**Western blott analysis to test de novo synthesis of non structural protien (NS5A) of HCV in lysates of infected Huh7.5 cells**. Huh7.5 cells were infected a pool of 20 HCV positive samples. Cells were lysed as described in Materials and Methods and the lysates were resolved on 12% SDS-PAGE, blotted onto nitrocellulose membranes and hybridized to anti-NS5A monoclonal antibody as primary antibody and the color was developed enzymatically with anti mouse IgG as secondary antibody. A distinct specific band of ~ 56 kDa corresponding to the NS5A protein appeared only in infected (lane 2) but not in the uninfected (lane 1) cells indicating de novo synthesis of viral non structural proteins following infection with the virus. Lane M represents the Protein marker (Blue Ranger Pre-stained Marker Mix; Pierce, U.S.A).

To examine whether infection of Huh7.5 cells with pooled HCV positive sera resulted in viral replication and de novo synthesis of non structural proteins, a mouse monoclonal antibody to NS5A protein was employed as a primary antibody to detect intracellular NS5A antigen within the resolved cell lysates. A distinct recognizable band at ~ 56 kDa (Figure 4) corresponding to newly synthesized NS5A protein was only detected in infected cells, indicating that these cells do support expression of viral non structural proteins.

### Testing the immuno-reactivity of caprine anti E2 Abs with Huh7.5 expression of E2 antigen

Three approaches were followed to test this immunoreactivity, namely western blot, immunocytochemistry and FACS analysis.

a) Western blot analysis of infected Huh7.5 cells using caprine anti p412 Ab to detect intracellular viral E2 antigens.

**Figure 5:**
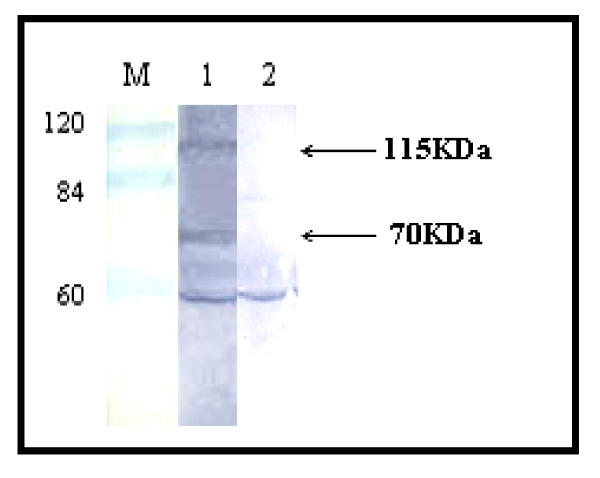
**Western blott analysis to test the efficacy of anti p412 caprine antibody to recognize the corresponding intracellular viral antigens**. Huh7.5 cells were infected with pooled HCV positive sera. Cell lysates were subjected to western blotting analysis using caprine anti p412 antibody. Two proteins of molecular weights 115kDa and 70kDa appeared only in HCV infected cells (lane 1) but not in uninfected cells (lane 2). M represents the Protein marker (Blue Ranger Prestained Marker Mix; Pierce, U.S.A).

To determine whether the generated caprine antibodies were able to react with intracellular E2 antigen as a structural protein, western blot hybridizations of HCV infected cell lysates were compared with lysates from uninfected cells. Caprine anti p412 Ab could detect the expression of two immunogenic proteins of molecular weights ~115 and 75 kDa that were not detectable in uninfected cells (Figure 5). Similar results were also obtained when using anti p430 and anti p517 Abs.

b) Intracellular HCV immunostaining assay

**Figure 6:**
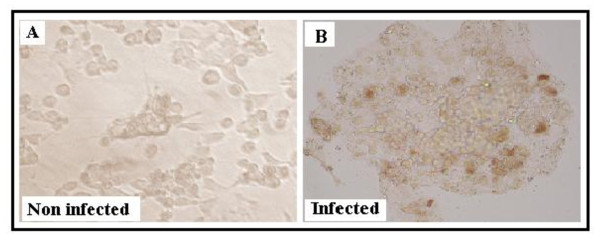
**Immunostaining of infected Huh7.5 cells with anti p412 caprine antibody**. Huh7.5 cell lines were infected with positive HCV sera as described in Materials and Methods. Immunostaining was carried out after 5 days from infection. Anti p412 was used as a primary antibody and anti-caprine peroxidase as a secondary antibody. DAB was used as substrate. Brown foci were visualized in infected cells (B) but not in non infected cells (A).

To confirm the observed immunoreactivity of caprine Abs in detecting the intracellular viral E2 proteins, either in a form of free antigen or on surface of viral particles, HCV infected or uninfected Huh7.5 cells were immunostained as described in Materials and Methods using anti p412 as a primary Ab. The results shown in Figure 6 indicate great specificity of caprine Abs where only infected cells contained distinct colored foci that were not detected in uninfected cells.

c) Flow Cytometric analysis of HCV E2 expression on surface of Huh7.5 cells using caprine anti p412 Ab.

**Figure 7:**
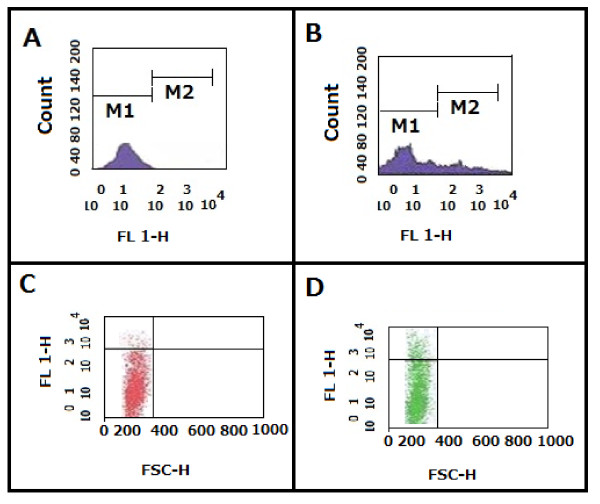
**A single parameter histogram for Flow Cytometric analysis of HCV E2 expression on surface of Huh7.5 cells using caprine anti p412 Ab**. Huh7.5 cells were infected with pooled HCV positive sera. After one week cells were harvested and incubated with caprine anti p412 Ab, washed and stained with FITC labeled anti goat Ab. Labeled cells were analyzed with flow cytometry (FACS Calibure, Becton Dickinson). Results showed that the mean fluorescence intensity of the infected Huh7.5 cells (B &D) was markedly higher (30%) than the uninfected Huh7.5 cells (A & C) demonstrating the specificity of anti p412 Ab to recognize the E2 protein on surface of HCV infected cells.

To test whether expressed E2 antigen in infected Huh7.5 cells was detectable by caprine Abs generated against E2 derived epitopes, surface staining of HCV E2 antigen in infected Huh7.5 cells was performed by indirect immuno-fluorescence. Cells were incubated with anti p412Ab, stained with FITC labeled antigoat Ab and analyzed with flow cytometer. Results in Figure 7 showed that the mean fluorescence intensity of the infected Huh7.5 cells was significantly higher than the uninfected Huh7.5 cells thus demonstrating the marked sensitivity and specificity of anti p412 Ab immunoreactivity towards E2 protein. Similar results were also obtained when either caprine anti p430 or p517 Abs were used indicating similar immunoreactive properties of all E2 derived Abs used in this study.

### Effect of caprine anti E2 Abs on HCV 4a RNA replication in Huh7.5 cells

**a) **Titration of Ab concentration achieving viral neutralization

**Figure 8:**
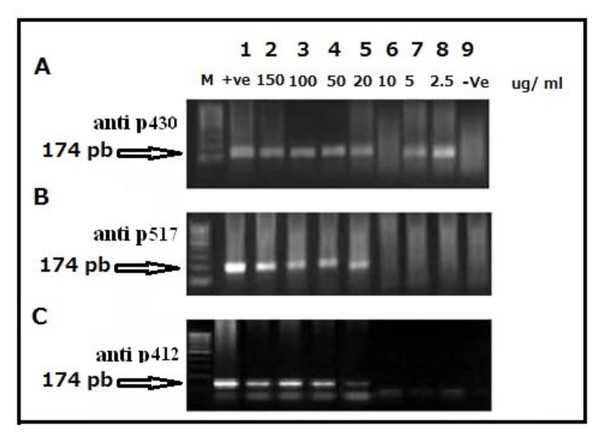
**Agarose gel electrophoresis of nested RT-PCR products to determine the neutralizing doses of the generated goat E2 antibodies**. The neutralizing dose of anti p517 and anti p412 started at 10 μgm/ml and continued to, as low as, 2.5 μgm/ml while the least and only neutralizing dose of anti p430 was 10 μgm/ml.

Testing the neutralizing activity of serial Ab concentrations (Figure 8) demonstrated that 10 μg/ml from anti p412 Ab had the highest neutralizing activity on HCV entry and replication.

**Figure 9:**
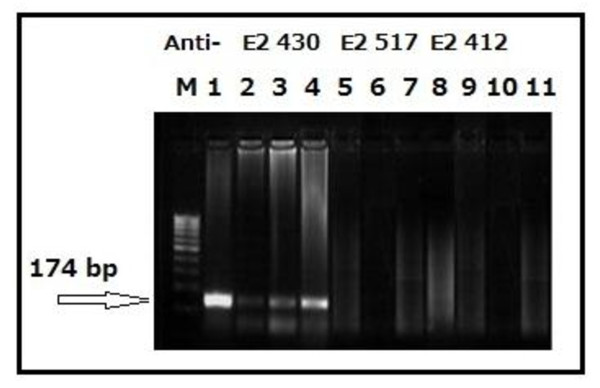
**Agarose gel electrophoresis of nested RT-PCR amplification of intracellular HCV RNA following incubation with decreasing concentrations of anti E2-epitopes caprine antibodies**. Huh7.5 cells were infected *Invitro *using a pool of 10 HCV genotype 4 positive sera with viral loads ranges (9 × 10^5^-1.5 × 10^6 ^copies/ml) in absence (lane 1) and presence (lanes 2-10) of serial dilutions (10 - 2.5 ug/ml) of caprine Abs. Cellular RNA was amplified using HCV 5'UTR specific nested primers as described in Materials and Methods. Agarose gel shows the status of Ab inhibition; anti p430 (A, lanes 2, 3, 4), anti p517 (B, lanes 5, 6, 7) and anti p412 (C, lanes 8, 9, 10). M is a molecular weight standard DNA marker (100 basepair; Jena Bioscience, Germany). Lane 11 shows the results of normal non infected Huh7.5 cells (negative control).

b) To test whether caprine Abs towards different E2 epitopes elicit the same blocking activity on HCV replication in Huh7.5 cells, the latter were infected separately with 20 HCV 4a positive sera, with varying values of viral loads, in absence and presence of 10 μg/ml of either caprine anti p430, anti p517 or anti p412 purified Abs in the culture medium. Intracellular HCV RNA was extracted, nested RT-PCR amplified and resolved on 2% agarose gel. The results show that this method exhibited 80% efficiency of viral infection into Huh7.5 cells. Furthermore, anti p517 and anti p412 Abs exhibited the highest potency in blocking of viral replication in cells (75% and 87.5% respectively) as compared with anti p430 Ab (31.5%). These results were confirmed by findings displayed in Figure 9 produced after infecting Huh7.5 cells with a pool of HCV 4a positive sera and incubation with the 3 tested caprine Abs at decreasing concentrations of purified Abs (10 - 2.5 μg/ml). Figure 9 showed that the tested range of anti p517 and anti p412 Ab concentrations did induce total inhibition of intracellular viral replication, whereas anti p430 Ab failed to induce inhibition of HCV replication at concentrations below 10 μg/ml.

c) Interference exerted by non neutralizing Abs on viral neutralization

**Figure 10:**
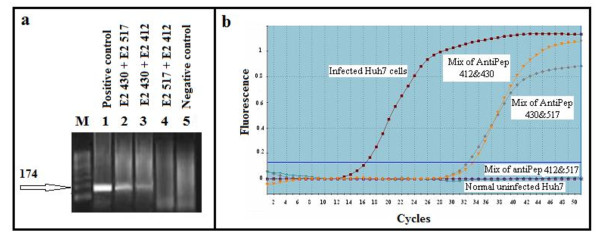
**Neutralizing activities of combinations caprine E2 antibodies as measured by RT-PCR and real time PCR**. Figure 10a Agarose gel electrophoresis of nested RT-PCR products of HCV 4a infected Huh7.5 cells to assess the optimum combinations of caprine E2 antibodies that block the viral infection into cells. Huh7.5 cells were infected *Invitro *using (virus + Ab) mixes each was made up of a pool of 10 HCV 4a positive sera (1.5 × 10^6^-3 × 10^6 ^copies/ml) mixed with different combinations of caprine anti p430, anti p517 and anti p412Abs. Cells were preincubated with virus/Ab mixes overnight at 4°C. Cells incubated with HCV pool serve as positive control; lane 1. Mixes containing anti p430 showed partial inhibition of intracellular viral replication (lanes 2 and 3). Total blockage of HCV RNA replication inside Huh7.5 cells was achieved by a combination of caprine anti p517 and anti 412 Abs (lane 4). Products of non-infected Huh7.5 cells as negative control were presented in lane 5). M is a molecular weight standard DNA marker (100 basepair; JenaBioscience, Germany). Figure 10b Qualitative evaluation of combined effect of goat E2 antibodies that block HCV infection *invitro*. The results obtained revealed that although the mix of goat anti peptides 430 & 517 and the mix of anti p430 & anti p412 didn't block the viral replication but yet it was delayed such that the amplification started at cycle 32 and cycle 33, respectively, instead of cycle 16 as it was shown for the infected Huh7.5 cells (positive control). On the other hand the mix of the goat anti p412 & anti p517 showed, similar results as in negative control uninfected Huh7.5 cells, without any significant amplification (below the cutoff line), indicating complete viral inhibition.

The possible steric hindrance of anti p430 Ab on the couple of neutralizing E2 Abs; namely anti p412 and anti p517 Abs were demonstrated in Figure 10(a) and 10(b) where anti p430 Ab induced significant interference with the viral neutralization capacities of both anti p412 and anti p517 Abs.

## Discussion

Three synthetic peptides were mapped to conserved epitopes within the carboxy terminal region of the HVR-1 of E2 protein. These immunogenic peptides were selected to be genetically conserved at least among viral subtypes infecting the local population, predominantly 4a. The peptide p430 showed 88% homology with HCV genotype 4a while p517 and p412 showed 93% and 100% homology respectively [16]. The present data demonstrate that immunization of caprines with these peptides can elicit significant humoral responses and the generated Abs were able to recognize relevant epitopes, either as the free soluble antigen or on surface of HCV particles in sera of chronically infected patients, thus suggesting its usefulness as specific diagnostic reagents. The expression of E2 related peptide in western blots at 58 kDa upon treatment with caprine IgG only in HCV positive sera confirms earlier data [24] that it represents a deglcosylated E2 protein and suggests the presence of immunodominant conserved epitopes within the E2 gp which encompass motifs of several linear epitopes. We have recently shown that these linear epitopes stimulated humoral and cellular responses in caprines [16] and in mice [25]. The observed immunoreactivity of these peptides with human IgG in HCV positive patients suggest that those E2 epitopes are immunogenic in humans, a hypothesis awaiting confirmation in future testing of healthy volunteers.

Hepatoma cells Huh7.5 posses CD81 receptor, a strong candidate receptor that enables HCV particles to bind to cells [26,27]. We have clearly demonstrated that direct infection of Huh7.5 cells from HCV positive sera resulted in viral entry into the cells as indicated by considerable quantities of intracellular viral RNA genomic and antigenomic strands throughout the duration of cell culture. The viral entry was followed by de novo synthesis of both structural and non structural proteins E2 and NS5A suggesting that these cells support the de novo expression of viral proteins. The appearance of two immunogenic peptides 70 and 115 kDa in infected Huh7.5 cells represent E2 protein and E1/E2 dimer respectively, thus supporting previous reports [28-30]. Since NS5A is not detectable in HCV positive sera its detection in cell lysates represents a marker for efficient infection and intracellular HCV replication [31]. Infected Huh7.5 cells were able to shed virus into culture medium, where the later could efficiently infect naïve Huh7.5 cells (results not shown). Infection of cells *in vitro *with HCV from a variety of sources can at least demonstrate the infectivity of the virus making these systems useful in evaluating drugs for antiviral activity or inhibition of HCV replication [19,22,32]. Transfection with synthetic full-length or near-full-length HCV RNA has the drawback that the RNA used for transfection is homogenously synthesized containing well defined sequence whereas in cases of natural infection with the whole viral particles several quasispecies of different magnitudes of infectivity could be coexisting, thus mimicking the *in vivo *infections of HCV. Other disadvantage of synthetic subgenomic virus transfection could be the absence of one or more non structural genes, which does not allow assembly of infectious viral particles as demonstrated by failure to infect chimpanzees [33]. When the full viral genome was put in frame downstream a strong promoter, it is obviously expected that the replication and the regulation of expression of viral proteins will not take place under viral regulatory elements. Besides, the system has the limitation of being restricted to single quasispecies and the chances of variant production, mimicking the natural infection, are poor.

In the present study we thought that not all antibodies raised against conserved peptides are neutralizing. Although caprine anti p430 Ab did not interfere with HCV entry at concentrations below 10 μg/ml, caprine anti p412 and p517 Abs, on the other hand, were able to block HCV entry into cells at concentration as low as 2.5 μg/ml, suggesting that the later two Abs are potentially neutralizing. The reason why high Ab concentrations did not neutralize the virus could attributed to aggregation and inability to bind to their corresponding epitopes, [34] a phenomenon previously referred to as zone tolerance between the amounts of Ab relative to its Ag. In general, the current data confirm our earlier results that caprine anti p412 and anti p517 Abs could bind HCV in immuncapture experiments while anti p430 Ab could not [16]. This agrees to earlier findings of Zhang *et al*., [11] who mapped two epitopes within a short segment of E2: epitope I, at amino acids 412-419 and epitope II, at amino acids 434-446 and demonstrated that epitope I, but not epitope II, was involved in virus neutralization under their experimental conditions, using HCV genotype 2a. The region encompassing amino acids 432-447 can be recognized by at least three monoclonal antibodies. Hsu *et al*., [35] using HCV pseudoparticle assay demonstrated that these MAbs were involved in viral neutralization. Knowing that genotypes 1, 4, 5, and 6 are serologically more closely related to each other than to genotypes 2 and 3 [36], the possibility that epitope II in various genotypes is associated with neutralization could not be excluded. Needless to say, assay systems and virus genotypes used in different studies may explain different outcomes of neutralization studies. To examine the possibility that anti p430 Ab displays a steric/or masking effect on the viral neutralization properties exerted by either anti p412 or anti p517 Abs, we performed viral neutralization assays in presence of a variety of Ab mixes. The current results clearly demonstrate that anti p430 Ab displayed a negative effect on the other two Abs so that it demolished their neutralizing activities, while a mixture of anti p412 and anti p517 exhibited potent viral neutralization properties either as singles or in combinations. These *in vitro *experiments perhaps explain the observed failure of viral neutralization and/or clearance in chronic HCV patients despite the presence of a pool of E2 Abs in their sera. These findings suggest that caprine anti p517 and anti p412 Abs may have been interfered by anti p430 Ab either at the level of viral adsorption and/or entry into cells thus leading to failure of inhibition of HCV replication inside the cells. This conclusion confirms the hypothesis made by von Hahn *et al*., [37], Stamataki et al., [38] and Zhang et al., [11] that co-existence of non neutralizing and neutralizing Abs may lead to hindrance of neutralizing activity in chronic HCV patients.

The reason for the failure of Huh7.5 infection by 4 out of 20 HCV positive sera is not clear; it is possible that free β-lipoproteins in human serum compete with the virus towards LDL receptors on surface of Huh7.5 cells [39]. We cannot neglect other reports claiming that the current in vitro system is not 100% permissible [40], and that the efficiency of viral infection could not be predictable for a given sample. Besides, the present data showed that no obvious correlation could be drawn between infectivity and viral RNA titer thus confirming early results of Rumin *et al*.,[41]. Although the two candidate neutralizing Abs i.e. anti p412 and anti p517 Abs displayed 87.5% and 75% viral neutralization efficiency (table 1), at least two possible explanations do exist for failure to inhibit viral infection in 12.5% and 25% of cases respectively; 1) the presence of escape mutants of viral isolates leading to failure to block mutants by caprine Abs thus allowing viral entry and replication and/or 2) over consumption of caprine Abs by un-assembled E2 protein released to serum from ruptured infected liver cells and varies in quantities from patient to patient [42].

**Table 1:** Efficiency of blocking intracellular viral replication by caprine anti E2 epitopes

Groups of HCV samples*		Number of samples (n = 20)	Viral load copies/ml (Mean ± S.D)
Successfully the cells	infected	16/20 (80%)	517,646 - 1,952,184
Successfully with anti p430	blocked	5/16 (31.25%)	313,992 - 940,408
Successfully with anti p517	blocked	12/16 (75%)	510,059 - 1,694,683
Successfully with anti p412	blocked	14/16 (87.5%)	497,516 - 1,985,120

* If the P-value is ≤ 0.05 the difference between the means is considered statistically significant.

T-test was conducted on all groups and results revealed that the viral load had no effect on the viral infectivity (*P *= 0.3786) in the two main groups, samples that failed to infect the Huh7.5 cells (n = 4) and samples that successfully infected the Huh7.5 cells (n = 16). Also, the viral load had no effect on the blocking activity of both generated anti p517 (p = 0.211) and anti p412 (p = 0.9285) but with anti p412 the viral load significantly affected the blocking activity of the generated caprine antibody (p = 0.0161).

Liver transplant recipients mostly suffer re-infection of grafts shortly after transplantation. One approach to control re-infection is the combined use of specific antiviral together with HCV-specific antibodies [43]. So we anticipate that the long term benefit of this study will be to generate fully human monoclonal antibodies against HCV E2 synthetic peptides derived from genetically conserved regions and use as supplement to IFN based therapy in post transplants of HCV positive patients. One could also predict that exclusive administration of neutralizing epitopes would be rather adequate to confer prophylaxis against early exposure to HCV infection. In conclusion, taking together the results of humoral immunity and cellular responses suggest that p412 and p517 peptides described herein are potential candidates for designing a prophylactic and/or therapeutic vaccine against HCV for further clinical studies.

## Competing interests

The authors declare that they have no competing interests.

## Authors' contributions

Part of these data was derived from YE PhD thesis. ME, AT and AH acted as supervisors on thesis. YE made alignments of E2 peptides, produced and purified antibodies and performed neutralization experiments. AT followed up all technical steps, wrote the draft of manuscript and partially subsidized the study. NE, AH followed up all technical steps. RM, RE carried out immunoassays. KA carried out real time PCR. ME designed the study, finalized the manuscript in its final form and partially subsidized the study. All authors read and approved the final manuscript
